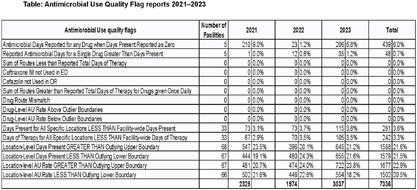# Analysis of Antimicrobial Use Quality Reports from the NHSN AU Option in Tennessee 2021–2023

**DOI:** 10.1017/ash.2025.244

**Published:** 2025-09-24

**Authors:** Dipen M Patel, Glodi Mutamba, Christopher Evans

**Affiliations:** 1 Tennessee Department of Health; 2 TN Department of Health

## Abstract

**Background:** The National Healthcare Safety Network (NHSN) Antibiotic Use (AU) Option aids hospital antimicrobial stewardship programs (ASPs) by facilitating tracking and reporting of AU data. In 2021, the Tennessee Department of Health (TDH) launched an AU data quality project to improve reporting accuracy. Quarterly reports are generated, assessing data across 15 quality flags, such as reporting antimicrobial days when days present (DP) are zero or drug-route mismatches. Flags also highlight significant outliers, including DP or AU rates outside the median ±2 interquartile ranges compared to the prior year. Reporting facilities receive actionable solutions for flagged concerns. **Method:** Data from AU quality flag reports generated by the NHSN AU Option for Tennessee facilities (2021–2023) were analyzed in this cross-sectional study. The analysis summarized the frequency and distribution of flagged issues across facilities and time. Archived data were utilized, excluding updates facilities made after quarterly reports. Quarterly flags per category were calculated for each facility, with total flags compiled annually to determine category frequency and percentage. Additionally, the number of distinct facilities contributing to the annual flag count was evaluated, providing insights into data quality trends across the study period. **Result:** From 2021 to 2023, 97 facilities submitted data to the NHSN AU Option, resulting in 7336 flags identified in the AU quality reports (Figure 1). The most frequent flag was “location-level AU rate greater than outlying upper boundary” (n=1677, 22.9%), reported by 67 facilities and the highest reported in 2023 (n=722, 23.8%). The second was “location-level DP greater than outlying upper boundary” (n=1588, 21.6%), reported by 68 facilities and highest in 2021 (n=547, 23.5%). The most frequent non-outlier-based quality issue was “antimicrobial days reported for any drug when DP were reported as zero” (n=439, 6.0%) followed by “antimicrobial days for a single drug greater than DP” (n=48) **Conclusion:** The study reveals data quality concerns in AU reporting among Tennessee facilities. Flags with changes in “Location-Level Days Present” and “AU Rate” outliers being prominent across the study period. These findings underscore the need for continuous monitoring and targeted feedback to enhance data accuracy, as well as a need for antimicrobial stewardship personnel to be able to identify and address changes in prescribing patterns and patient populations efficiently within their facilities. Addressing recurring challenges identified can improve AU data reliability, supporting more effective antimicrobial stewardship and better patient care outcomes.

**Figure:**